# Supplementary material for: Mechanisms governing the pioneering and redistribution capabilities of the non-classical pioneer PU.1
Source: Nat Commun. 2020 Jan 21;11:402. doi: 10.1038/s41467-019-13960-2 (PMC6972792; doi:10.1038/s41467-019-13960-2)
Supplement: Supplementary file 7 — Source data [file 41467_2019_13960_MOESM7_ESM.zip › Source_Data/FigureS3/FigureS3H_MotifScanOutput/homerResults/motif2.similar.html]

motif2

## Information for motif2

A
T
C
G
T
C
G
A
G
T
A
C
C
G
T
A
A
C
T
G
A
C
T
G
C
G
T
A
C
G
T
A
C
T
A
G
A
G
C
T
T
A
C
G
C
T
A
G
  
Reverse Opposite:  

G
A
T
C
A
T
G
C
T
C
G
A
A
G
T
C
C
G
A
T
A
C
G
T
A
G
T
C
A
G
T
C
A
C
G
T
A
C
T
G
A
G
C
T
T
A
G
C
  

|  |  |
| --- | --- |
| p-value: | 1e-286 |
| log p-value: | -6.589e+02 |
| Information Content per bp: | 1.652 |
| Number of Target Sequences with motif | 1019.0 |
| Percentage of Target Sequences with motif | 26.36% |
| Number of Background Sequences with motif | 2907.0 |
| Percentage of Background Sequences with motif | 6.49% |
| Average Position of motif in Targets | 245.5 +/- 241.8bp |
| Average Position of motif in Background | 206.9 +/- 139.8bp |
| Strand Bias (log2 ratio + to - strand density) | -0.0 |
| Multiplicity (# of sites on avg that occur together) | 1.10 |
| Motif File: | file (matrix) reverse opposite |

### Similar de novo motifs found

|  |  |  |  |  |  |  |  |
| --- | --- | --- | --- | --- | --- | --- | --- |
| Rank | Match Score | Redundant Motif | P-value | log P-value | % of Targets | % of Background | Motif file |
| 1 | 0.964 | T A G C T C G A T G A C C G T A A C T G A C T G C G T A C G T A C T A G A G C T A C T G | 1e-282 | -651.391380 | 28.92% | 7.94% | motif file (matrix) |
| 2 | 0.980 | T C G A G A T C G C A T C A G T A G T C T A G C C A G T A C T G A G C T | 1e-266 | -612.591832 | 29.67% | 8.80% | motif file (matrix) |
| 3 | 0.960 | C T G A A G T C C G A T A C G T A G T C A G T C A C G T A C T G | 1e-243 | -560.112674 | 38.46% | 15.18% | motif file (matrix) |
| 4 | 0.943 | T A G C C T G A A G T C C G A T C A G T A T G C T G A C A C G T T A C G G A C T | 1e-237 | -546.901202 | 28.63% | 8.99% | motif file (matrix) |
| 5 | 0.789 | C T G A T G A C C G T A C T A G C T A G C G T A G C A T C T A G A G C T A C T G A T C G A C G T C G A T | 1e-198 | -457.452522 | 10.61% | 1.19% | motif file (matrix) |
| 6 | 0.914 | C G T A A T C G T C G A T G A C C G T A A C T G A C T G C G T A C G T A T C G A C G A T A C T G T A C G A C G T | 1e-188 | -433.096040 | 13.97% | 2.56% | motif file (matrix) |
| 7 | 0.860 | T G A C C G A T T A G C G C T A T A G C C G T A A C T G C T A G G T C A C G T A C T A G A T G C G T A C T C G A | 1e-101 | -233.117164 | 21.62% | 9.44% | motif file (matrix) |
| 8 | 0.913 | G A C T A T G C G T A C C T G A A G C T A G C T C G A T A G T C G T A C A C G T A C T G G C A T C T A G | 1e-100 | -231.128808 | 12.05% | 3.52% | motif file (matrix) |
| 9 | 0.836 | T A G C G T C A T A G C C G T A A C T G C T A G G T C A C T G A C T G A G A T C G T A C G C T A A T G C | 1e-78 | -180.124100 | 12.13% | 4.27% | motif file (matrix) |
| 10 | 0.801 | C G T A T G A C G C T A C A T G C T A G T G C A C T G A T C A G C A G T C A T G T A C G C A G T | 1e-74 | -172.036435 | 31.89% | 18.91% | motif file (matrix) |
| 11 | 0.808 | G T A C G C T A T A C G T C G A T G A C G C T A T A C G A T C G G T C A C T G A T C G A C A G T T A C G | 1e-68 | -158.232547 | 8.64% | 2.59% | motif file (matrix) |
| 12 | 0.821 | C T A G T A C G T G A C C G T A C T A G A T C G G T C A C T G A T C A G T G A C C T A G T C A G T C A G | 1e-45 | -104.958505 | 12.26% | 5.82% | motif file (matrix) |
